# Micro‐Topographies Induce Epigenetic Reprogramming and Quiescence in Human Mesenchymal Stem Cells

**DOI:** 10.1002/advs.202203880

**Published:** 2022-11-22

**Authors:** Steven Vermeulen, Bart Van Puyvelde, Laura Bengtsson del Barrio, Ruben Almey, Bernard K. van der Veer, Dieter Deforce, Maarten Dhaenens, Jan de Boer

**Affiliations:** ^1^ Department of Instructive Biomaterials Engineering MERLN Institute University of Maastricht Maastricht 6229 ER The Netherlands; ^2^ Department of Biomedical Engineering and Institute for Complex Molecular Systems Eindhoven University of Technology Eindhoven 5600 MB The Netherlands; ^3^ Laboratory of Pharmaceutical Biotechnology Department of Pharmaceutics Ghent University Ghent 9000 Belgium; ^4^ Laboratory for Stem Cell and Developmental Epigenetics Department of Development and Regeneration KU Leuven Leuven 3000 Belgium

**Keywords:** biomaterials, epigenetics, mechanobiology, mesenchymal stem cells, nucleus

## Abstract

Biomaterials can control cell and nuclear morphology. Since the shape of the nucleus influences chromatin architecture, gene expression and cell identity, surface topography can control cell phenotype. This study provides fundamental insights into how surface topography influences nuclear morphology, histone modifications, and expression of histone‐associated proteins through advanced histone mass spectrometry and microarray analysis. The authors find that nuclear confinement is associated with a loss of histone acetylation and nucleoli abundance, while pathway analysis reveals a substantial reduction in gene expression associated with chromosome organization. In light of previous observations where the authors found a decrease in proliferation and metabolism induced by micro‐topographies, they connect these findings with a quiescent phenotype in mesenchymal stem cells, as further shown by a reduction of ribosomal proteins and the maintenance of multipotency on micro‐topographies after long‐term culture conditions. Also, this influence of micro‐topographies on nuclear morphology and proliferation is reversible, as shown by a return of proliferation when re‐cultured on a flat surface. The findings provide novel insights into how biophysical signaling influences the epigenetic landscape and subsequent cellular phenotype.

## Graphical abstract

Biomaterials can profoundly affect the size and shape of the nucleus, yet the phenotypical consequences of these alterations are still largely unknown. In this work, the effect of nuclear confinement on mesenchymal stem cell behavior is elucidated through histone mass‐spectrometry and microarray datasets. Here, a reduction of ribosomal and nucleoli proteins and histone acetylation is connected with a quiescent phenotype.

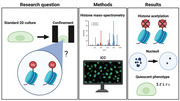

## 1 Introduction

Primary cells exposed to a standard in vitro culture environment can quickly lose morphological characteristics and phenotypic marker expression, as is well described for chondrocytes and tenocytes in Refs. [1, 2]. This phenomenon also occurs for multipotent cells, such as mesenchymal stem cells (MSCs), which coincides with a gradual decrease of multipotency after multiple passages.^[^ ^3^ ^]^ This cell culture characteristic impedes tissue‐engineering applications since tissue or organ construction requires that cells retain the proper phenotype and can be grown in sufficiently large numbers. Different biochemical approaches exist to alleviate this problematic phenomenon. For example, the addition of growth factors improves MSC multipotency,^[^ ^4^ ^]^ while coating the culture surface with matrix compounds promotes the phenotypic maintenance of hepatocytes.^[^ ^5^ ^]^ Next to controlling the biochemical environment, biomaterials are an exciting focus for directing cell fate, which have gained popularity for cell culture applications in recent years.

Biomaterials exert physical stimuli on cells that mimic essential aspects of the physiological environment, allowing control over cell behavior. Examples include the wide variety of surface structures such as grooves and topographies in micro‐and nano‐dimensions that both regulate differentiation processes in stem cells,^[^ ^6^, ^7^, ^8^, ^9^, ^10^ ^]^ maintenance of their stemness,^[^ ^11^, ^12^, ^13^, ^14^, ^15^ ^]^ and the phenotypical maintenance of primary cells.^[^ ^2^, ^16^, ^17^ ^]^ Furthermore, biomaterials such as microwells, scaffolds or hydrogels that introduce cells into 3D environments, induce similar effects.^[^ ^18^, ^19^, ^20^, ^21^ ^]^ Therefore, it is evident that biomaterials are valuable tools for controlling the cell culture environment. Nevertheless, how biomaterials elicit their biological effects is unclear and requires further investigation to optimize and improve biomaterial design.

In recent years, multiple studies found that numerous biomaterial‐induced mechanisms elicit mechanobiological responses in cells. Examples include altered cadherin signaling through cell–cell contacts^[^ ^22^ ^]^ or changes in integrin‐mediated signaling through cell–substrate interactions.^[^ ^23^, ^24^, ^25^, ^26^ ^]^ An important mechanobiological factor in this regard are changes in cell morphology and cytoskeletal organization, as shown by experiments where inhibiting cytoskeletal‐related signaling abolishes the mechanobiological effects of biomaterials.^[^ ^27^, ^28^, ^29^, ^30^ ^]^ Although cytoskeletal‐related signaling can be attributed to the Rho/ROCK/SRF^[^ ^27^, ^30^, ^31^, ^32^ ^]^ and the YAP/TAZ pathway,^[^ ^33^, ^34^, ^35^ ^]^ an interesting observation is that changes in cell morphology coincides strongly with nuclear morphology,^[^ ^36^, ^37^, ^38^ ^]^ making it difficult to distinguish if physical cues exert their effects through changes in either cell or nuclear morphology. Nevertheless, research does indicate that altering nuclear morphology can directly influence cell behavior.^[^ ^39^ ^]^ Furthermore, nuclear shape alterations are associated with epigenetic changes, such as chromatin organization or histone modifications.^[^ ^40^, ^41^ ^]^ This is an interesting concept considering that histone acetylation and methylation determine gene expression levels, is altered during embryonic stem cell and MSC differentiation, and has thus a unique signature for each cell type.^[^ ^42^, ^43^, ^44^, ^45^ ^]^ Therefore, it is imperative to investigate how biomaterials affect the epigenetic landscape in order to fundamentally understand the phenotypical consequences that biomaterials elicit.

Previous research demonstrates that various biomaterial types can alter nuclear morphology, chromatin architecture, and histone modifications. For example, changing the chemical composition or the stiffness of the substrate alters nuclear shape and chromatin architecture.^[^ ^46^, ^47^ ^]^ Concerning histone modifications, introducing epithelial cells into a 3D culture decreases histone acetylation,^[^ ^41^ ^]^ with similar effects observed with smaller adhesive islands compared to larger adhesive islands.^[^ ^48^ ^]^ Surface structures such as microgrooves allow increasing histone acetylation,^[^ ^49^ ^]^ which is associated with improved fibroblast reprogramming toward induced pluripotent stem cells (iPSCs),^[^ ^50^ ^]^ emphasizing the relevance of understanding the epigenetic consequences of biomaterials on cell phenotype.

In this study, through state‐of‐the‐art histone mass‐spectrometry, we demonstrate that also micro‐topographies,^[^ ^51^ ^]^ of which we previously established a relationship between design features and nuclear shape,^[^ ^52^ ^]^ influences the epigenetic landscape in MSCs. Through microarray and proteomics data, we now provide an extensive investigation into micro‐topographical‐induced epigenetic alterations. Furthermore, we discovered that micro‐topographies affect nucleoli architecture, which is strongly affected by nuclear confinement. To our knowledge, this is a novel observation that warrants further investigation since nucleoli architecture is associated with disease states such as viral infection and cancer.^[^ ^53^ ^]^ Here, we associate these phenomena with a quiescent phenotype which supports previous observations where we found a decrease in proliferation and metabolism of MSCs cultured on micro‐topographies.^[^ ^37^ ^]^ These findings provide novel mechanobiological insights into how biomaterials influence the phenotype of a cell through altering nuclear morphology.

## 2 Results

### 2.1 Micro‐Topographies Elicit a Broad Diversity in Nuclear Confinement and Deformation

**Figure 1:**
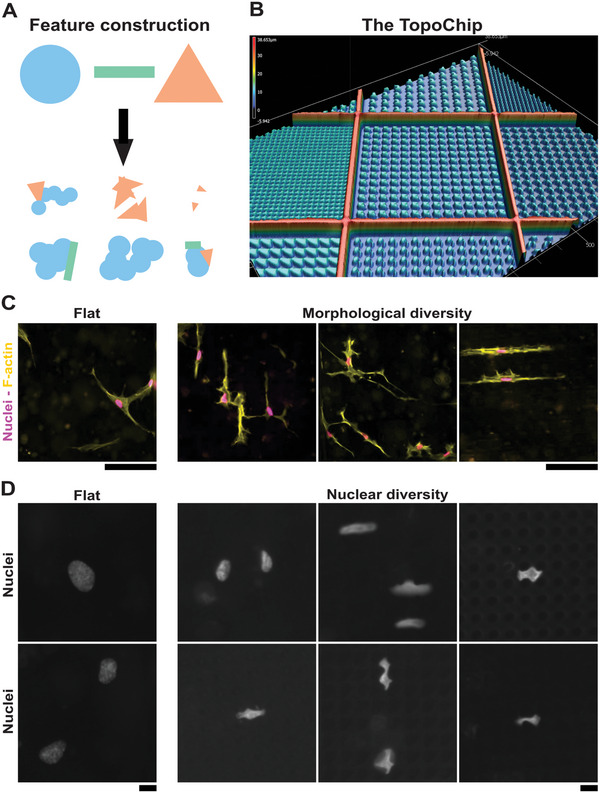
Micro‐topographies create a large variety in cell‐ and nuclear morphological characteristics. A) Through a circle, rectangle and triangle, we constructed the design space of the TopoChip. B) Profilometric image of the TopoChip platform. A 2 × 2 cm^2^ chip with 2176 unique micro‐topographies with a height of 10 µm and walls of 30 µm. C) Illustration of morphological diversity elicited by micro‐topographies. Depicted are MSCs cultured on the flat control surface and three micro‐topographical surfaces. F‐actin was stained with phalloidin conjugated with AlexaFluor‐568. Nuclei were counterstained with Hoechst 33258. Scale bar represents 100 µm. D) Representative images of nuclear deformation occurring in MSCs elicited by micro‐topographies. Depicted are nuclei of MSCs cultured on the flat control surface and random micro‐topographies. In general, micro‐topographies elicit smaller nuclei, with different degrees of elongation and branching. Scale bar represents 10 µm.

It is well established that physical cues alter cellular morphological characteristics,^[^ ^28^, ^54^, ^55^, ^56^ ^]^ including those elicited by topographical structures.^[^ ^51^, ^57^, ^58^ ^]^ However, despite the strong relationship between cell and nuclear shape, the effect of physical cues on nuclear architecture has not yet been thoroughly investigated. We, therefore, aimed to investigate how different physical cues in micro‐topographical dimensions affect nuclear morphology. For this, we utilized the TopoChip,^[^ ^51^ ^]^ of which 2176 different surface topographies were created in silico by using three primitives in unique combinations (**Figure** **1**A). Before seeding MSCs onto the TopoChips, we assessed the quality of the substrate using profilometric imaging. This allowed confirming the spatial dimensions of the topographical features and the walls separating the individual units (Figure 1B). After a positive quality assessment, MSCs were seeded on eight TopoChips and cultured for 48 h, after which F‐actin and DNA were fluorescently labeled (Figure 1C). Since the TopoChip platform contains four flat surfaces and each micro‐topography in duplicate, a total of 32 flat surfaces and 16 replicates of each micro‐topography were included in the analysis. High‐content imaging of each unit enabled the identification of 41 540 cells. We observed a broad cellular morphological heterogeneity between the individual units, similar to previous TopoChip studies involving tenocytes^[^ ^2^ ^]^ and MSCs.^[^ ^59^ ^]^ Furthermore, we found a diversity of nuclear morphologies as well, with varying degrees of size and deformation (Figure 1D).

**Figure 2:**
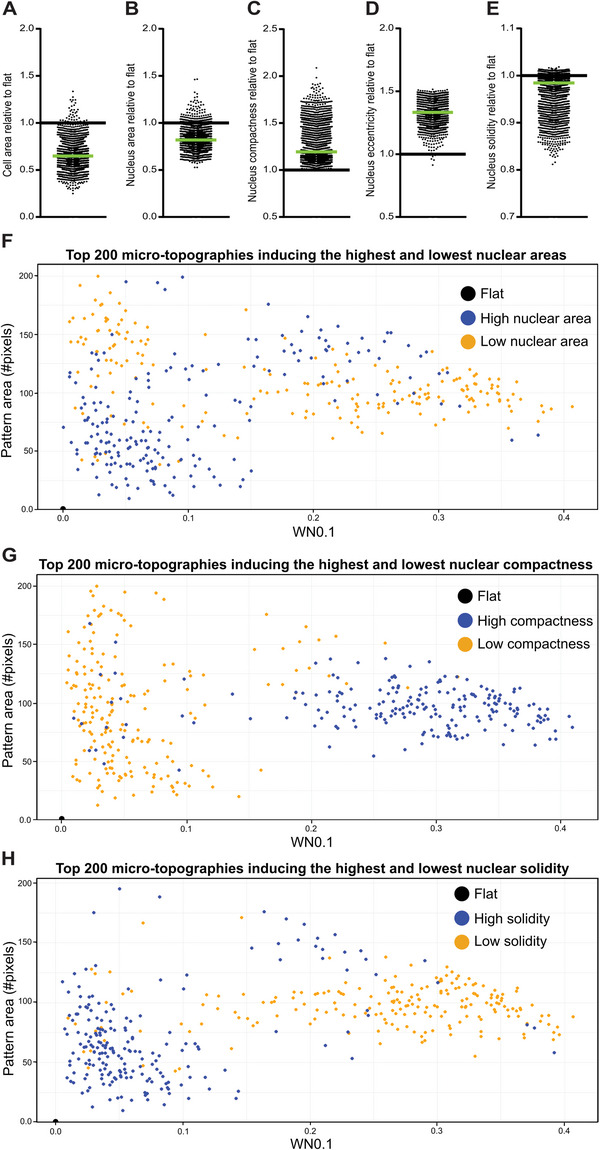
Surface parameters pattern area and WN0.1 promote nuclear deformation. A–E) Micro‐topographies induce a spectrum of different cell and nuclear sizes, elongation (compactness and eccentricity) and branching (solidity), with flat residing on the far side of the spectra. Each dot represents the median quantified values from an individual micro‐topography (*n* = 2176). The black line represents the flat surface as a reference, and the green line represents the median of all individual values. F) A large pattern area generally promotes a smaller nuclear size. An exception was found with surface patterns exhibiting a high pattern area and intermediate WN0.1, characteristic of high‐density patterns. G,H) Increased elongation (increased compactness) and branching (lower solidity) was found on micro‐topographies with intermediate pattern areas and high WN0.1, characteristic for surfaces that allow spacing in between the micro‐topographies. High nucleus/compactness/solidity; *n* = 200, low nucleus/compactness/solidity; *n* = 200.

Next, we quantified this diversity by measuring nuclear morphological characteristics through CellProfiler.^[^ ^60^ ^]^ Both cell and nucleus area were decreased for the majority of MSCs grown on the micro‐topographies, with 984 out of 2176 micro‐topographies (45%) inducing significant lower cell areas (**Figure** **2**A) and 580 out of 2176 micro‐topographies (26%) inducing significant lower nuclear areas (Figure 2B). In addition, by quantifying the morphological parameters’ compactness and eccentricity, we found that micro‐topographies often elicited nuclear elongation. We observed that 1692 micro‐topographies (77%) induced significantly higher compactness levels, while 1670 micro‐topographies (76%) induced significantly higher eccentricity levels compared to a flat surface (Figure 2C,D). Besides elongation, we were interested in determining nuclear branching or deformation by measuring solidity levels. 982 micro‐topographies (45%) induced significantly lower solidity levels and thus induced nuclear deformation (Figure 2E). We provide the reader in Figure S1, Supporting information, a visual explanation of the morphological parameters. These findings demonstrate that micro‐topographical diversity elicits diversity in nuclear morphology.

Next, we characterized the relationship between the feature parameters of the micro‐topographies and morphological outcomes. A complete overview of the surface parameters, such as pattern area, the number of primitives, or feature size, is documented in Table S1, Supporting Information, while all morphological parameters as output from CellProfiler can be found in Table S2, Supporting Information. We employed random forest algorithms to create models that associate nuclear morphological parameters with the feature descriptors. We used 75% of the lowest 200 and 75% of the highest 200 scoring TopoUnits associated with a morphological characteristic. The accuracy of the model was assessed on the remaining 25%. Models were trained with 10‐fold cross‐validation. When assessing which feature characteristics induced either low or high nuclear areas, we found that pattern area, the fraction covered by primitives (FCP) and wavenumber 0.1 (WN0.1), a Fourier transformation algorithm, were important determinants for the prediction model, with an area under the curve (AUC) of 94%, and an accuracy of 93% (Figure S2, Supporting Information). Plotting the WN0.1 and pattern area of micro‐topographies eliciting the smallest and largest nuclear areas indeed allows a separation between the top and lowest 200 micro‐topographies (Figure 2F). In general, an intermediate pattern area is associated with decreasing the size of the nucleus. However, an exception are cells cultured on dense patterns and an intermediate WN0.1, which indicates micro‐topographies where cells have little space to settle between the structures. A low WN0.1 and high pattern area decreases nuclear size, indicating that the distance between the features allows for cellular adaptation of their morphology. Similarly, plotting the WN0.1 and pattern area of micro‐topographies inducing the highest and lowest compactness and solidity levels (Figure 2G,H) illustrate that these surface feature parameters are most important for influencing them. Of interest is that WN0.1 can achieve this separation, independent of pattern area, indicating that the distance between the features is essential to achieve elongation and deformation of the nuclei.

These results demonstrate that the micro‐topographical design space of the TopoChip allows for choosing design parameters that precisely control nuclear shape parameters.

### 2.2 Micro‐Topographical Diversity Allows Controlling Histone 3 Acetylation Levels

**Figure 3:**
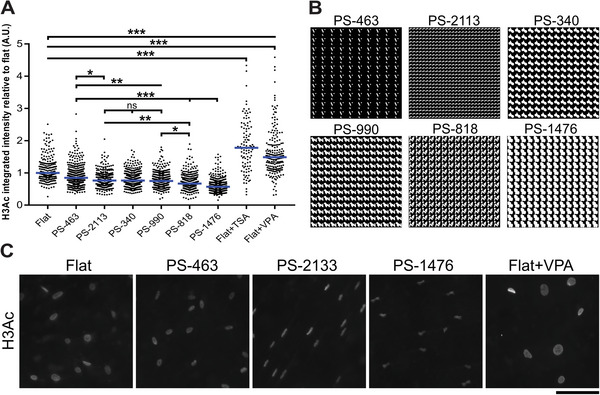
H3 acetylation levels can be regulated by micro‐topographical dimensions. A) Quantification of immunolabeled H3 acetylation on six different micro‐topographies reveals different levels of histone acetylation in MSCs. Surface PS‐463 elicits the least difference in acetylation levels compared to a flat surface, while PS‐1476 elicits the strongest reduction. No statistical significant difference was observed between PS‐2133, PS‐340, and PS‐990. HDAC inhibitors TSA and VPA induce elevated acetylation levels (* *p* < 0.05; ** *p* < 0.01; *** *p* < 0.001). Each dot represents individual nuclei. The blue line represents the median. Flat; *n* = 246, PS‐463; *n* = 583, PS‐2113; *n* = 322, PS‐340; *n* = 479; PS‐990; *n* = 388, PS‐818; *n* = 422, PS‐1476; *n* = 458, Flat+PSA; *n* = 99, Flat+VPA; *n* = 205. B) In silico design of the micro‐topographies. PS‐463 contains patterns with a low pattern area. PS‐1476 contains patterns with a high pattern area. C) MSCs cultured on PS‐463 exhibit smaller nuclei compared to MSCs on flat, yet with the same round morphology. Nuclei of MSCs grown on PS‐2133 exhibit smaller and elongated morphology, with PS‐1476 inducing the smallest and deformed nuclei. Scale bar represents 50 µm.

Since the diverse feature patterns on the TopoChip elicit multiple nuclear morphologies, we were interested if we could control histone acetylation, known to be influenced by biomaterials such as microgrooves and adhesive islands,^[^ ^41^, ^50^ ^]^ depending on the micro‐topography employed. We selected six different topographies with diverse parameters concerning pattern density and space between the pillars, seeded MSCs on them, and immunolabeled histone 3 (H3) acetylation after 48 h of culture. We also included the compounds trichostatin A (TSA), and valproic acid (VPA) in a flat culture as a reference. Both are histone deacetylase (HDAC) inhibitors and increase histone acetylation levels.^[^ ^61^, ^62^ ^]^ Intensity level quantification demonstrated a general decrease in H3 acetylation across the micro‐topographies (**Figure** **3**A). As expected, VPA and TSA increased acetylation levels in MSCs. The design of these surfaces is illustrated in Figure 3B, where we see that surface PS‐463 exhibits features with a low pattern area, while PS‐2113, PS‐340 and PS‐990 exhibit intermediate pattern areas. High‐density patterns are present on surface PS‐818 and PS‐1476, which coincided with the lowest acetylation levels in MSCs. Visual inspection of H3 acetylation reveals that this patterned area strongly influences nuclear area and that the lowest acetylation levels are found in the smallest nuclei (Figure 3C). Previous results established a correlation between cell/nuclear size and proliferation,^[^ ^10^, ^59^ ^]^ which we now further relate with histone acetylation. These results demonstrate that histone acetylation levels can be controlled through varying micro‐topographical pattern density.

### 2.3 Histone Mass‐Spectrometry Analysis of MSCs Cultured on the PS‐1018 Surface

**Figure 4:**
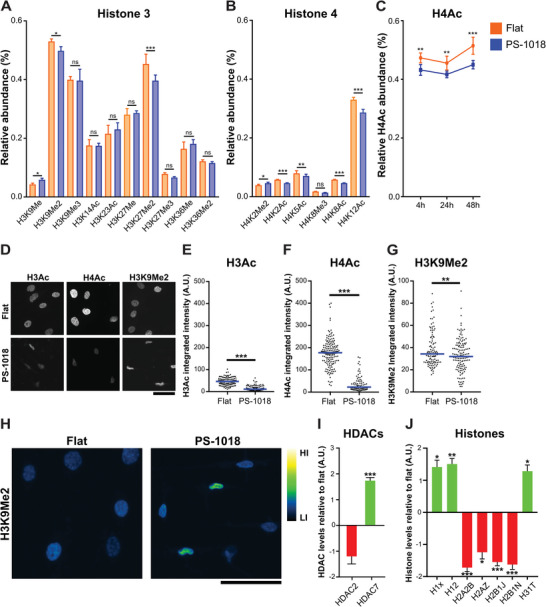
Histone mass‐spectrometry and immunostaining reveal a decline in histone acetylation for MSCs cultured on micro‐topography PS‐1018. A) Histone mass‐spectrometry performed on H3 reveals an H3K9Me/Me2 ratio increase and a decrease in H3K27Me2 when MSCs are cultured on the PS‐1018 surface after 48 h (*n* = 6). B) Histone mass‐spectrometry performed on H4 reveals a general decrease in acetylation levels, with a slight increase in H4K2 dimethylation. Bar plot represents mean with error bars representing SD (*n* = 6). C) Global H4 acetylation levels are already decreased 4 h after seeding on the PS‐1018 surface. Acetylation levels slightly increase after 48 h for MSCs cultured on both the flat and PS‐1018 surface compared to the 4 and 24 h time point (*n* = 6). D) Immunolabeled images of global H3 and H4 acetylation and H3K9Me2 of MSCs cultured on flat and PS‐1018 after 48 h. Scale bar represents 50 µm. E–G) Intensity quantification reveals a general decrease in H3, H4 acetylation levels, and H3K9Me2. Each dot represents individual nuclei. H3Ac; flat: *n* = 104, PS‐1018: *n* = 129. H4Ac; flat: *n* = 167, PS‐1018: *n* = 89 H3K9Me2; flat: *n* = 106, PS‐1018: *n* = 133. The blue line indicates the median of all values. H) Immunostaining of H3K9Me2 reveals a heterogeneous intensity pattern when MSCs are cultured on the PS‐1018 surface. H3K9Me2 levels of MSCs exhibiting elevated intensity levels are more located at the nuclear periphery. HI = high intensity levels, LI = low intensity levels. Scale bars represent 50 µm. I) Mass spectrometry revealed a 1.73‐fold increase in HDAC7 levels for MSCs cultured on PS‐1018. Bar plot represents mean with error bars representing SEM (*n* = 6). J) Histone and histone linker levels are altered by the decrease in nuclear size. Bar plot represents mean with error bars representing SEM (*n* = 6) (* *p* < 0.05; ** *p* < 0.01; *** *p* < 0.001).

After determining that micro‐topographies influences nuclear morphology, we investigated how this affects the global histone landscape. As a surface material, we selected the PS‐1018 surface, which we can fabricate in a 100 mm dish format, enabling us to generate sufficient biological material for downstream experiments. We previously utilized this surface for other research purposes,^[^ ^7^, ^10^, ^29^, ^37^, ^63^ ^]^ allowing us to build an in‐depth understanding of how this design affects cell biology. This surface induces elongated morphology in MSCs and a substantial reduction in nuclear area (Figure S3, Supporting Information). We therefore consider this an ideal model system to represent how micro‐topographies can change histone modifications. 48 h after cell seeding, we performed histone mass‐spectrometry on the chromatin fraction. We were able to detect ten histone modifications on the H3 tail (**Figure** **4**A) and six histone modifications on the histone (H4) tail (Figure 4B), both tails are considered to be the most extensively modified compared to histone 1 (H1) and histone 2 (H2).^[^ ^64^ ^]^ On the H3 tail, we found an increase for H3K9Me, a decrease for H3K9Me2 and H3K27me2, with no observed difference in H3K9Me3 between MSCs cultered on flat and PS‐1018. These observations are associated with alterations in transcriptional activity.^[^ ^65^, ^66^ ^]^ Concerning H3 acetylation, no differences were detected for H3K14Ac and H3K23Ac between MSCs cultured on flat and PS‐1018. This indicates that the observed differences in H3 acetylation from immunostaining experiments is the result from undetectable histone mass‐spectrometry H3 acetylation sites. The majority of significant differences were measured on the H4 tail, where acetylation levels were decreased for H4K2Ac, H4K5Ac, H4K8Ac and H4K12Ac, all indicative of chromatin compaction.^[^ ^67^ ^]^ For H4K2Me2, we also observed higher dimethylation levels. Next to a 48 h time point, we also quantified histone modifications at 4 and 24 h. When quantifying the total amount of H4 acetylation, we noticed that the decreased levels already become apparent 4 h after cell seeding (Figure 4C). After 48 h, we noticed an increase in global H4 acetylation on the flat and PS‐1018 surface. Nevertheless, H4 acetylation remained lowest throughout all time points on the PS‐1018 surface.

Due to the observed decrease in multiple H4 acetylation sites, we were interested in exploring the difference between acetylation staining intensities between the H3 and H4 tail, especially since not all H3 acetylation sites were detected with histone mass‐spectrometry. For this, we employed immunostaining with antibodies targeting global acetylation on the H3 and H4 tail while targeting H3K9Me2 as extra validation of the mass‐spectrometry data (Figure 4D). Through immunostaining, we found a global decrease in H3 acetylation intensity levels when MSCs are cultured on the PS‐1018 surface, as seen on previously investigated surfaces (Figure 4E). A similar observation was apparent for H4 acetylation levels, of which a more substantial decrease was observed compared to H3 acetylation (Figure 4F). For H3K9Me2, we found a slight decrease in intensity levels (Figure 4G). The heterogeneous H3K9Me2 intensity levels between MSCs cultured on the PS‐1018 surface are of interest, on which we also observed that some MSCs exhibit high H3K9Me2 intensity levels at the nuclei periphery, as seen through a heat map visualization (Figure 4H), indicating altered interaction dynamics between chromatin and the nuclear periphery.^[^ ^68^ ^]^ This observation indicates that next to histone modifications, the structural organization of chromatin is an important characteristic that changes upon exposure to micro‐topographies. Next to histones, the acid extraction method also co‐precipitated other proteins, thereby allowing their detection through mass spectrometry. Although representing an incomplete fraction of the proteome, interesting differences in protein abundancies are revealed between MSCs cultured on flat and PS‐1018. For example, concerning HDACs, we could detect HDAC2 and HDAC7 of which the latter showed a statistically significant 1.73‐fold increase in MSCs cultured on the PS‐1018 surface (Figure 4I). Of interest, HDAC7 is known to regulate H3K27Ac and might therefore be contributing to the decreased H3 acetylation levels we observe.^[^ ^69^ ^]^ Next to histone modifications, we also observed differences in histones and histone linker proteins. Specifically, we observed a decrease of histone H2A type 2‐B (H2A2B; −1.71 fold change), H2A histone family member Z (H2AZ;−1.24 fold change), histone H2B type 1J (H2B1J; −1.54 fold change) and histone H2B type 1‐N (H2B1N;−1.62 fold change), and an increase for linker histone H1× (1.41 fold change), linker histone H1‐2 (1.5 fold change) and histone H31T (1.28 fold change). In general, these observations demonstrate that micro‐topographical induced nuclear confinement coincides with a decrease in histone acetylation and reorganization of nucleosome constituents.

### 2.4 Micro‐Topographies Induce Chromatin Condensation and a Reduction of Nucleoli and Ribosomal Protein Abundancy

**Figure 5:**
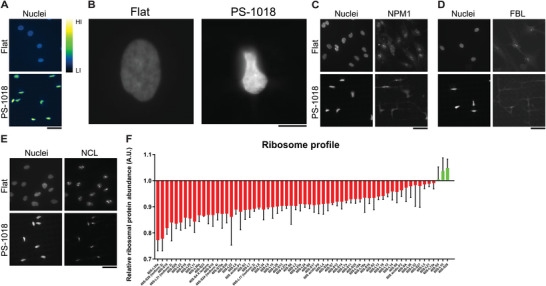
Micro‐topographies induce chromatin condensation and a reduction in nucleoli and ribosomal protein abundance. A) Heat map representation of Hoechst 33258 intensity levels reveals that the PS‐1018 surface induces chromatin condensation. HI = high intensity levels, LI = low intensity levels. B) Hoechst staining reveals strong chromatin compaction in the nuclei. Scale bar represents 10 µm. C–E) Immunostaining of the nucleoli markers nucleophosmin, fibrillarin, and nucleolin revealed a decrease in nucleoli when MSCs were cultured on the PS‐1018 surface. Nuclei counterstained with Hoechst 33258. Scale bar represents 50 µm. F) Proteomics data reveals a decrease in ribosomal protein abundance in MSCs cultured on the PS‐1018 surface (*n* = 3).

After assessing that micro‐topographies induce nuclear confinement, which coincides with profound reductions in global acetylation levels, we were interested in determining how other nuclear structures are affected. Foremost, we visualized chromatin condensation by creating a heat map of the Hoechst 33258 signal intensity on the PS‐1018 surface (**Figure** **5**A‐B). We found increased chromatin condensation as expected with the lower acetylation levels we observed.^[^ ^70^ ^]^ Next, since nucleoli play important roles in various biological functions, we wanted to investigate if micro‐topographies influence these nuclear components. For this, we immunolabeled the nucleoli for nucleophosmin (NPM1), which plays a function in essential biological processes such as ribosome biogenesis,^[^ ^71^ ^]^ mRNA processing,^[^ ^72^ ^]^ and chromatin remodeling.^[^ ^73^ ^]^ We also immunolabeled fibrillarin (FBL), a ribonucleoprotein involved in pre‐rRNA processing and ribosome assembly,^[^ ^74^ ^]^ and nucleolin (NCL), a nucleolar protein that is also associated with pre‐rRNA processing^[^ ^75^ ^]^ and chromatin condensation.^[^ ^76^ ^]^ Of interest, when comparing the immunostainings between flat and surface PS‐1018, we found a reduction in the number of nucleoli for all immunostainings, with the most profound effects visible for NPM1 and FBL (Figure 5C‐E). Due to the involvement of the nucleoli in ribosome biogenesis through rRNA transcription, we investigated if we could detect a reduction of ribosomal proteins in MSCs cultured on the PS‐1018 surface compared to flat. Through proteomics data retrieved from regular protein mass‐spectrometry, we indeed detected a decrease in the majority of ribosomal proteins (Figure 5F). Mass spectrometry applied to the histone protein fraction validated this general decrease of ribosomal proteins (Table S3, Supporting Information). In the histone protein fraction, we could also detect a decrease of NPM1 (−5.27 fold change) in MSCs cultured on the PS‐1018 surface compared to flat after 48 h of cell culture (Figure S4, Supporting Information). These findings indicate sharp alterations in nucleoli function, warranting further investigation, and is, to our knowledge, the first observation of a biomaterial inducing such a biological response in cells.

### 2.5 Micro‐Topographies Induce a Distinct Transcriptomic Profile Associated with Chromosome Organization and the Nucleolus

**Figure 6:**
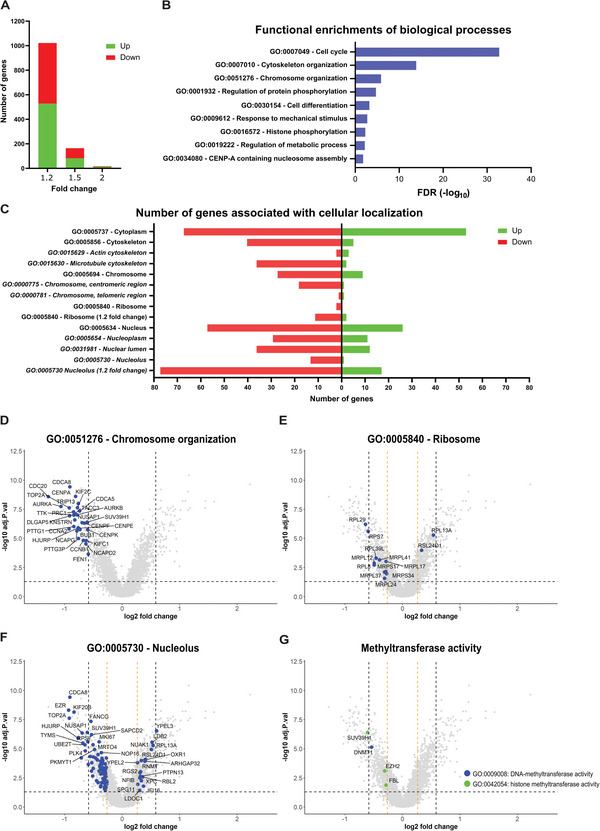
Micro‐topographies alter the gene expression profile of genes associated with the cell cycle, chromosome organization, ribosomes, and the nucleolus. A) Number of genes down‐or upregulated of MSCs cultured on surface PS‐281 compared to a flat surface. B) Functional protein enrichment analysis of biological processes. C) Overview of up‐ and downregulated genes associated with cellular localization. D–G) Volcano plot representation of genes associated with chromosome organization, the ribosome, the nucleolus, and methyltransferase activity. Highlighted in blue or green are genes associated with these GO terms. DEG cut‐off is determined at a 1.5 fold change (blue dashed line) or 1.2 fold change (orange dashed line) and an adjusted *p*‐value of 0.05.

Next, we searched our transcriptomics data library of MSCs cultured on micro‐topographies for additional information regarding epigenetic regulation. For this, we selected data from surface PS‐281, which exhibit similarities with the PS‐1018 surface concerning morphological characteristics such as nuclear deformation and elongation (Figure S5, Supporting Information). When MSCs were cultured on surface PS‐281, we found that 528 genes with a fold change of more than 1.2 were upregulated, and 492 genes were downregulated compared to a flat surface (**Figure** **6**A). Here, 82 genes were upregulated, and 82 genes were downregulated with a fold change of at least 1.5. Further restricting the fold change cut‐off to two, leads to twelve upregulated and five downregulated genes.

Generating a functional protein association network through STRING (https://string‐db.org/), revealed strong functional enrichments of biological processes (Figure 6B) associated with the cell cycle (false discovery rate [FDR] = 1.77.10^−33^), cytoskeletal organization (FDR = 1.35.10^−6^), chromosome organization (FDR = 1.35.10^−6^), regulation of protein phosphorylation (FDR = 1.94.10^−5^), cell differentiation (FDR = 0.00062), response to mechanical stimulus (FDR = 0.0018), histone phosphorylation (FDR = 0.0051), regulation of metabolic process (FDR = 0.0069) and centromere protein‐A (CENP‐A) containing nucleosome assembly (FDR = 0.0160). These results are not surprising considering we previously demonstrated that micro‐topographies decrease proliferation,^[^ ^10^ ^]^ strongly affect cytoskeletal organization,^[^ ^29^ ^]^ while altering the differentiation potential of MSCs.^[^ ^59^ ^]^ Of interest, we found a strong overlap in genes associated with the GO terms cell cycle, cytoskeletal and chromosome organization (Figure S6, Supporting Information), further emphasizing a connection between cell and nuclear size and the epigenetic modifications we observe.

Next, we counted the amount of up‐ and downregulated genes based on the cellular localization of their associated proteins (Figure 6C). As shown previously, most cytoskeleton genes were downregulated,^[^ ^29^ ^]^ with most belonging to the microtubule cytoskeleton. This coincides with a reduction of genes located at the nucleus, chromosome, and nucleolus. When also taking genes up or downregulated with a fold change of at least 1.2 into account, we found an even greater increase of downregulated genes associated with the ribosome and nucleolus. This is in line with the reduction of nucleoli and ribosomal proteins we observed (Figure 5C‐F).

To gain further insight into how micro‐topographies influence chromosome organization, we investigated which genes are associated with the GO term “chromosome organization” (Figure 6D). A list of these genes is located in Table S4, Supporting Information. Here, we found a downregulation of 41 genes and an upregulation of one gene associated with this GO term. In the list of the downregulated genes, we found aurora kinase A (AURKA; −2.09 fold change) and AURKB (−1.71 fold change), both proteins located near the centrosome where they are involved in chromosome movement and organization.^[^ ^77^, ^78^ ^]^ We also detected a downregulation of multiple centromere proteins, including CENPA (−1.80 and −1.70 fold change), CENPE (−2.01 fold change), CENPF (−1.57 fold change), CENPK (−1.51 fold change) and CEMPM (−1.53 fold change). These proteins are specialized H3 variants located at the centrosome, associated with unique chromatin regions distinct from traditional euchromatin and heterochromatin.^[^ ^79^ ^]^ Related to this, we observed a downregulation of Holliday junction recognition protein (HJURP; −1.7 fold change), which is involved in incorporating CENPA in centromeres.^[^ ^80^ ^]^ We also detected a downregulation of the histone‐modifying enzyme suppressor of variegation 3–9 homolog 1 (SUV39H1; −1.52 fold change), which might be involved in the lower H3K9Me2 levels we observed^[^ ^81^ ^]^ (Figure 4A). We further found a downregulation of protein regulator of cytokinesis 1 (PRC1; −1.78 fold change), known for its role in playing a crucial role in cytokinesis.^[^ ^82^ ^]^

Concerning genes associated with the ribosome, we found a downregulation of eleven and the upregulation of two ribosomal proteins with a fold change of at least 1.2 (Figure 6E and Table S5, Supporting Information). In this list, the majority are mitochondrial ribosomes such as mitochondrial ribosomal protein L12 (MRPL12; −1.4 fold change) and mitochondrial ribosomal protein L41 (MRPL41;−1.3 fold change) involved in metabolic activity. In general, this suggests that the reduction of ribosomal protein synthesis might be partially mediated through reduced transcription. For genes associated with the nucleolus, we found a downregulation of 77 genes and an upregulation of 13 genes (1.2 fold change cut‐off) (Figure 6F and Table S6, Supporting Information), further confirming the ICC findings that nucleoli function is altered on micro‐topographies. Although we observe the presence of ribosomal genes and genes associated with the chromosome in this list, we also mention the downregulation of Nucleolar Protein 7 (NOL7; −1.23 fold change) and Nucleolar Protein 11 (NOL11; −1.24 fold change), both nucleolar‐associated proteins that are involved in proper rRNA processing.^[^ ^83^ ^]^ Focusing on specific histone methyltransferase activity (Figure 6G and Table S7, Supporting Information), we observed a downregulation of the previously mentioned SUV39H1 (−1.52 fold change), but also for Enhancer of Zeste Homolog 2 (EZH2; −1.23 fold change), and FBL (−1.21 fold change). Enhancer of Zeste Homolog 2 (EZH2) catalyzes the addition of methyl groups to histone H3 at lysine 27, and might, therefore, also be implicated in the reduction of H3K27Me2 we observed (Figure 4). Of interest, the downregulation of FBL further supports the ICC observations (Figure 5). In light of this, we also mention the downregulation of NCL (−1.31 fold change), while NPM1 shows no differences in gene expression between flat and PS‐281 (data not shown). Considering that we also do not detect alterations in HDAC genes on the transcriptional level, these observations demonstrate that the presence of these proteins might be controlled  independent of transcriptional regulation. Next to histone methyltransferase activity, we also found a reduction of (cytosine‐5)‐methyltransferase 1 (DNMT1; −1.45 fold change). Although this protein is associated with DNA methylation, it is also involved in the methylation of H3K9.^[^ ^84^ ^]^ These examples demonstrate that the nuclear deformation that micro‐topographies elicit coincides with an altered gene expression profile involved in chromosome organization, nucleoli and ribosomal protein abundance, and epigenetic alterations.

### 2.6 Micro‐Topographies Induce a Quiescent‐Like Phenotype in MSCs

**Figure 7:**
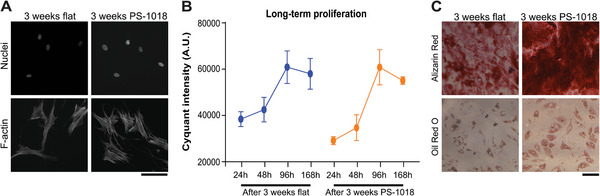
Micro‐topographies allow a reversible phenotype in MSCs, yet retain multipotency. A) MSCs cultured for 3 weeks on micro‐topography PS‐1018 reversibly adapt their spindle‐shaped morphology back toward a spread‐out morphology when cultured again on a flat surface. B) MSCs cultured for 3 weeks on PS‐1018 retain normal proliferation speeds when cultured again on a flat surface. C) MSCs demonstrated a higher degree of multipotency when cultured on micro‐topographies for 3 weeks compared to MSCs cultured on a flat surface. Scale bars represent 100 µm.

Our previous findings suggest a relationship between decreased nuclear size and reduction in metabolism^[^ ^37^ ^]^ and proliferation.^[^ ^10^, ^59^ ^]^ Furthermore, the reduction of chromosome and cell cycle‐related genes, nucleoli, ribosomal proteins, and histone acetylation levels observed in this study further builds upon the case that micro‐topographies induce a quiescent‐like phenotype. Because stemness is associated with quiescence, we were interested in assessing if micro‐topographies promote the maintenance of multipotency of MSCs. Foremost, we wanted to determine if the phenotypical changes that micro‐topographies elicit are permanent or reversible when MSCs are cultured back on a flat surface. Establishing this is important for understanding the implications of MSC culture on micro‐topographies and how these structures can subsequently be utilized for tissue engineering applications. For this, we cultured MSCs for three weeks on the PS‐1018 surface, after which we reseeded them on a flat surface. We found that MSCs cultured from the PS‐1018 surface on a flat surface retained a spread‐out morphology, indistinguishable from MSCs that were solely cultured on a flat surface (**Figure** **7**A). Subsequently, we tested if proliferation could return to normal levels. We found that MSCs, after three weeks of culture on the PS‐1018 surface, had a similar proliferation speed as MSCs cultured for three weeks on a flat surface (Figure 7B). To assess multipotency, we seeded MSCs for three weeks on the PS‐1018 surface, after which we seeded them on a flat surface and assessed their potential to differentiate toward the osteogenic and adipogenic lineage compared to cells cultured for three weeks on a flat surface. Of interest, we found that MSCs cultured for an extended period on the PS‐1018 surface had an improved differentiation potential, as shown by an increased presence of mineralization and fat deposits, compared to MSCs cultured on a flat surface for the same period (Figure 7C). These findings suggest that micro‐topographies induce a quiescent‐like phenotype in MSCs and prolong multipotency in cell culture.

## 3 Discussion

Our study demonstrates that micro‐topographies alter nuclear size, affect histone acetylation, nucleoli abundance, and both chromosomal‐associated and histone‐modifying gene expression levels in human MSCs. This is caused by the confinements imposed on the nuclei by many of the topographies. In our experiments, histone acetylation is reduced by topographies, a finding aligned with reports in the literature in which nuclear confinement, induced by other biomaterial types, reduces histone acetylation. For example, decreased cell area on adhesive islands is associated with lower histone acetylation.^[^ ^48^ ^]^ Also, 3D cultures reduce nuclear size and lower acetylation levels.^[^ ^41^ ^]^ These observations are extendable beyond physical cues, for example, changing the chemistry on the surface leads to a reduction in nuclear area and subsequent chromatin compaction,^[^ ^46^ ^]^ while altering stiffness levels reduces nuclear area and acetylation.^[^ ^47^ ^]^ This contrasts with another study where mouse embryonic fibroblasts cultured on micro‐grooves show elevated histone acetylation levels.^[^ ^50^ ^]^ In the context of that study, an interesting follow‐up study is to investigate if the reprogramming efficiency of fibroblasts toward iPSCs is hindered on our micro‐topographies due to lower acetylation levels. It can be concluded that biomaterials profoundly impact nuclear architecture, and further research is needed to elucidate the biological impact they elicit. In this regard, the observation that micro‐topographies can fine‐tune acetylation levels is interesting for cell culture applications that require specific histone acetylation signatures in cells. For example, to induce a specific histone acetylation signature that corresponds to a particular cell phenotype. As such, we might be able to predict based on the nuclear deformation a biomaterial elicits what the outcome will be of stem cell differentiation. We do emphasize however, that follow‐up research is required such as chromatin immunoprecipitation (ChIP)‐sequencing to determine which genes are influenced by the differences in acetylation levels.

A novel finding of our work is the reduced presence of nucleoli when cells are cultured on micro‐topographies. Nucleoli are responsible for rRNA processing and subsequent ribosome synthesis. The number of nucleoli is directly associated with proliferation since cell division requires sufficient ribosomal components for protein synthesis. This supports our hypothesis that micro‐topographies induce a quiescent‐like phenotype characterized by a loss of cell cycle progression. It would be interesting to follow‐up if other biomaterial types that induce cell confinement elicit similar alterations and subsequent phenotype. How physical cues initiate the pathways behind this phenomenon is subject to future investigation. Applications that can arise from this concept can be the long‐term maintenance of cell phenotype, as demonstrated in this study by the maintenance of MSC multipotency. Others have shown before that other biomaterials, such as disordered nano‐topographies, can achieve similar effects in MSCs.^[^ ^14^ ^]^ Here, it would also be interesting to verify if this effect is associated with the epigenetic alterations found in this article. Lower acetylation levels are associated with a more naïve state, such as in embryonic stem cells (ESCs),^[^ ^85^, ^86^ ^]^ which is also known to coincide with reduced proliferation speed.^[^ ^87^ ^]^ Therefore, growing sufficient cell numbers for tissue generation might be challenging with a micro‐topographical cell culture. However, the quiescent‐like phenotype that our micro‐topographies elicit is very relevant for tissue‐engineering applications that require such a phenotype. Since MSCs can control the behavior of other cells through paracrine signaling, micro‐topographies could be interesting for co‐culture applications or the harvest of cell media for clinical applications after sufficient secretion of growth factors.^[^ ^88^ ^]^ Also, the concept can also be applied when cells inside a biomaterial implant are required to secrete soluble factors for improved tissue healing.^[^ ^89^ ^]^

Although we used state‐of‐the‐art histone mass‐spectrometry, other techniques can further elucidate the biological implications that micro‐topographies elicit on the epigenetic landscape. For example, ChIP‐sequencing can provide information on which specific parts of the genome are affected by the reduced acetylation and other modifications we measured. Furthermore, it would be interesting to investigate how micro‐topographies affect chromatin folding and structure. Since we observed higher intensity levels of H3K9Me2 on the nuclear periphery, utilizing techniques that identify specific DNA regions that come into contact with the nuclear periphery can give us more insights into the structural organization of the genome when cells are cultured on micro‐topographies.^[^ ^68^ ^]^ Also, other techniques, such as high‐throughput chromosome conformation capture (Hi‐C) or advanced scanning electron microscopy (SEM) imaging, can allow us to gain additional insights into this organization.^[^ ^90^, ^91^ ^]^ In the future, utilizing these techniques for micro‐topographies and other surface structures will help gain additional insights into how biomaterials affect the epigenetic landscape.

## 4 Experimental Section

### Surface Fabrication

A detailed description of the surface fabrication procedures can be found elsewhere.^[^ ^92^ ^]^ In short, the inverse pattern of the topographies was etched into a silicon wafer by directional reactive ion etching (DRIE). To facilitate demolding procedures, the wafer was coated with a layer of trichloro(1H,1H,2H,2H‐perfluorooctyl)silane (FOTS, Sigma‐Aldrich). Polydimethylsiloxane (PDMS; Down Corning) was cured on the silicon wafer to generate a positive mold and was subsequently used as a template to create a second negative mold in Ormostamp polymer (micro resist technology Gmbh), which serves as the mold for hot embossing the polystyrene (PS) films (Goodfellow). The hot embossing procedure was carried out at 140 °C for 5 min, and a pressure of 10 Bar, with a demolding temperature of 90 °C. Before cell culture, the PS topographies and the flat PS surface were treated with oxygen plasma to improve cell adhesion for 30 s at 0.8 mbar, 50 sccm O_2_ and 100 W, which lowered the contact angle (CA) of the surfaces (Figure S7, Supporting Information). Quality of the fabricated imprints was assessed using a Keyence VK‐H1XM‐131 profilometer. Chemical analysis of the TopoChip surfaces was performed previously through time‐of‐flight secondary ion mass spectrometry (ToF SIMS) and X‐ray photoelectron spectroscopy (XPS) analysis.^[^ ^93^ ^]^

### Cell Culture

Adipose‐derived human mesenchymal stem cells (AD‐hMSCs) used in this study were purchased from Lonza. AD‐hMSCs were isolated with consent from a 42‐year‐old female. The studies involving human participants were reviewed and approved by the Medical Ethical Committee (METC) of the Maastricht University Medical Center (#15‐4‐274). Basic medium for AD‐hMSCs consists of MEM Alpha GlutaMAX, no nucleosides (Gibco). Basic media was supplemented with 10% v/v fetal bovine serum (FBS; Sigma), 0.2 mm ascorbic‐acid‐2‐phosphate (ASAP), and 10 U/mL Penicillin/Streptomycin (ThermoFisher). Cells were grown in a humid atmosphere at 37 °C and 5% CO_2_.

### Microarray Study

Bone marrow‐derived human MSCs were seeded on topography PS‐281 for 24 h in basic medium at a density of 15 000 cells/cm^2^ in 24 well plates in three replicas. Total RNA was isolated using the Nucleospin RNA isolation kit (Macherey–Nagel). Then, from 100 ng of RNA, cRNA was synthesized using the Illumina TotalPrep RNA amplification kit. Both RNA and cRNA quality was verified on a Bioanalyzer 2100 (Agilent). Microarrays were performed using Illumina HT‐12 v4 expression Beadchips. 750 ng of cRNA was hybridized on the array overnight, after which the array was washed and blocked. Then, through the addition of streptavidin Cy‐3, a fluorescent signal was developed. Arrays were scanned on an Illumina Beadarray reader, after which raw intensity values were background corrected in BeadStudio (Illumina). Further data processing and statistical testing were performed using the online portal arrayanalysis.org. The probe‐level raw intensity values were quantile normalized and transformed using variance stabilization (VSN). A detection threshold of 0.01 was used for reducing the number of false positives. A linear modeling approach with empirical Bayesian methods, as implemented in Limma package, was applied for differential expression analysis of the resulting probe‐level expression values. *p*‐values were corrected for multiple testing using the Benjamini and Hochberg method. Genes with a corrected *p*‐value below 0.05 were considered differentially expressed. GO gene lists were obtained from AmiGO 2. A protein‐protein interaction map and false discovery rates of functional enrichments were established by applying the DEG list with a 1.5 fold change as cut‐off value in STRING version 11.5. Minimal interaction score to establish the network was set at 0.4.

### Histone Isolation and Propionylation

Adipose‐derived MSCs were seeded on flat and PS‐1018 surfaces and cultured for 4, 24, 48, and 72 h in basic medium at a density of 15 000 cells/cm^2^ in a 6‐well plate format. Cells were pooled together, after which the cell pellet was flash frozen. This process was repeated 5 times to obtain 6 replicates. Histone extraction was performed using a direct acid protocol as described elsewhere.^[^ ^94^ ^]^ Therefore, 2 million cells were resuspended in 250 µL of 0.4 n HCl and incubated for 4 h on a rotator at 4 °C. The histones were precipitated with 33% trichloroacetic acid (TCA) while incubated on ice for 30 min. Afterward, the precipitated histones were washed twice with ice cold acetone to remove residual TCA. A small amount of the extracted histone fraction, corresponding to 200.000 cells was quantified by gel‐electrophoresis using an 8–16% TGX gel (Biorad) and a commercially available bovine histone standard (Roche). The remaining purified histones of each sample were vacuum dried and propionylated.^[^ ^95^, ^96^ ^]^ In short, histones were dissolved in 20 µL 1 m triethylammonium bicarbonate (TEAB) buffer (Sigma‐Aldrich), pH 8.5 before 20 µL of propionylation reagent (1:79 v/v of a propionic anhydride and 2‐propanol mixture) was added, for an incubation of 30 min at room temperature. This was followed by adding 20 µL milliQ water (Merck Millipore) for 30 min at 37 °C. Histones were then digested overnight at 37 °C using trypsin (Promega) at an enzyme/histone ratio of 1:20 m/m in 500 mm TEAB, supplemented with CaCl_2_ and ACN to a final concentration of 1.0 mm and 5%, respectively. Subsequently, the propionylation reaction was carried out again to cap peptide N‐termini. Overpropionylation at serine, threonine, and tyrosine was reversed by resuspending the vacuum dried sample in 50 µL 0.5 m NH_2_OH and 15 µL NH_4_OH at pH 12 for 20 min at room temperature after which 30 µL pure formic acid was added. Finally, the samples were vacuum dried again and stored for LC‐MS analysis.

### Liquid Chromatography and Mass Spectrometry Analysis

The samples were resuspended in 0.1% Formic Acid (FA) in water to achieve a histone concentration of 200 ng µL^−1^. Internal standards Beta‐Galactosidase (B‐gal) (Sciex) and MPDS2 (Waters) were spiked in a concentration of 12.5 fmol µL^−1^. Quality control (QC) samples were made by mixing 1 µL of each biological sample. Histones were analyzed by micro‐RPLC‐MS using a nanoACQUITY UPLC system (Waters) coupled to a Synapt G2‐Si Q‐TOF (Waters) mass spectrometer. An ACQUITY Symmetry C18 180 µm × 20 µm trap column (Waters) was combined with an ACQUITY M‐Class CSH C18 300 µm × 100 mm column (Waters) at a flow rate of 5 µL min^−1^ (0.1% FA spiked with 3% DMSO). A 90 min gradient from 3–40% ACN in 0.1% FA was applied with a total scan time of 105 min per sample. The sample list was randomized and interspersed with QC injections. Each cycle consisted in one full MS1 scan (m/z 50–5000) of 200 ms, followed by an MS2 scan of 100 ms. The 10 most intense precursor ions were selected from a single MS survey scan for MS2 fragmentation.

### Histone Mass Spectrometry Data Analysis

Data analysis was done as described earlier.^[^ ^97^ ^]^ The .raw data was imported and aligned in Progenesis QI for Proteomics (QIP) 4.1 (Nonlinear Dynamics, Waters) before feature detection was performed. For identification, the five tandem MS2 spectra closest to the elution apex were selected for each feature and exported as .mascot generic format file (.mgf) to search with Mascot 2.6.1 (Matrix Science). The obtained .mgf was searched using three types of searches: a) a standard search for the identification of non‐propionylated histones and to validate the presence of the internal standards B‐gal and MPDS2; b) an error tolerant search to identify all the proteins in the sample; and c) multiple sequential searches with six different PTM sets for the identification of modified histone peptides. The different PTM sets were selected using an established concept, to reduce the ambiguity in annotation. The following parameters were set in all three searches: two missed cleavages, peptide mass tolerance of 10 ppm, and a fragment mass tolerance of 50 ppm. For search a) trypsin was set as digestion enzyme, while ArgC (only cleaves after arginine residues) was set for the error tolerant search (b) and the sequential searches (c). Next, the identification files were exported as .xml files and imported in Progenesis QIP. Manual validation of the annotated histone features was performed to resolve isobaric near‐coelution. Afterward, the same procedure for identification was repeated and finally these identification results were also imported in Progenesis QIP.

### Protein Mass Spectrometry In‐Liquid Digestion

A total of 60 µg protein in 50 µL 50 mm ammonium bicarbonate (ABC) with 5 m urea was used. 5 µL of dithiothreitol (DTT) (20 mm final) was added and incubated at room temperature for 45 min. The proteins were alkylated by adding 6 µL of IAA solution (40 mm final). The reaction was taken place at room temperature for 45 min in the darkness. The alkylation was stopped by adding 10 µL of DTT solution (to consume any unreacted IAA) and incubated at room temperature for 45 min. For the protease digestion, 2 µg trypsin/lysC was added to the protein and incubated at 37 °C for 2 h. 200 µL of 50 mm ABC was added to dilute the urea concentration and further incubated at 37 °C for 18 h. The digestion mix was centrifuged at 2 × 10^3^ g for 5 min and the supernatant collected. Samples were subsequently labeled with TMT isobaric mass tagging labelling reagent (10‐plex; Thermo Scientific) according to the manufacturer's protocol. In short, 60 µg of protein for each sample was used. The TMT labelling reagents were dissolved in 41 µL acetonitrile per vial. The reduced and alkylated samples and control were added to the TMT reagent vials. The reaction was incubated for 1 h at room temperature and quenched for 15 min by adding 8 µL of 5% hydroxylamine. Equal amounts of the samples and control were combined in a new vial and analyzed by liquid chromatography‐tandem mass spectrometry (LC‐MS/MS).

### Protein Identification Using LC‐MS/MS

A nanoflow HPLC instrument (Dionex ultimate 3000) was coupled on‐line to a Q Exactive (Thermo Scientific) with a nano‐electrospray Flex ion source (Proxeon). The final concentration of the TMT labeled digest/peptide mixture was 0.33 µg µL^−1^ and 5 µL of this mixture was loaded onto a C18‐reversed phase column (Thermo Scientific, Acclaim PepMap C18 column, 75‐µm inner diameter × 15 cm, 2‐µm particle size). The peptides were separated with a 90 min linear gradient of 4–68% buffer B (80% acetonitrile and 0.08% formic acid) at a flow rate of 300 nL min^−1^. MS data was acquired using a data‐dependent top‐10 method, dynamically choosing the most abundant precursor ions from the survey scan (280–1400 m/z) in positive mode. Survey scans were acquired at a resolution of 70 × 10^3^ and a maximum injection time of 120 ms. Dynamic exclusion duration was 30 s. Isolation of precursors was performed with a 1.8 m/z window and a maximum injection time of 200 ms. Resolution for HCD spectra was set to 30 000 and the Normalized Collision Energy was 32 eV. The under‐fill ratio was defined as 1.0%. The instrument was run with peptide recognition mode enabled, but exclusion of singly charged and charge states of more than five.

### Immunocytochemistry

After cell culture, cells were washed with phosphate‐buffered saline (PBS; Merck) and fixed with 4% w/v paraformaldehyde (Sigma‐Aldrich) for 5 min at 37 °C. After a washing step, cells were permeabilized with 0.01% v/v Triton X‐100 (Acros Organics) and blocked with goat serum (1:100; Sigma‐Aldrich) in PBT (PBS + 0.02% Triton‐X‐100, 0.5% BSA) for 1 h. Afterward, cells were incubated with the primary antibody in PBT for 1 h. After a washing step, cells were incubated with a secondary antibody conjugated to an Alexa Fluor (1:500; ThermoFisher) in PBT. In conjunction, phalloidin conjugated to an Alexa Fluor (1:500; ThermoFisher) in PBT was added to the sample for 1 h. After washing, the nucleus was counterstained with Hoechst 33258 (1:1000; Sigma‐Aldrich) for 10 min. After a subsequent washing step, surfaces were mounted on glass cover slides with mounting media (Dako). All washing steps were performed in triplicate with PBT. Primary antibodies used in this study are: anti‐acetyl‐histone H3 antibody (1:200; Millipore; 06–599), anti‐acetyl histone H4 antibody (1:200; Abcam; ab177790), anti‐H3K9Me2 (1:200; Abcam; ab1220), anti‐nucleophosmin antibody (1:200; Abcam; ab37659), anti‐fibrillarin (1:100; Abcam; ab4566), and anti‐nucleolin antibody (1:200; ThermoFisher; ZN004).

### Image Analysis

Fixed samples were inverted, and fluorescent images were acquired through a glass coverslip using a fully automated Nikon Eclipse Ti‐U microscope in combination with an Andor Zyla 5.5 4MP camera. Fluorescent images were analyzed through CellProfiler 3.1.8,^[^ ^60^ ^]^ applying custom‐made pipelines. After illumination corrections, the morphology of the nucleus was captured by the Otsu adaptive thresholding method applied on the Hoechst 33258 image channel. Subsequently, cell morphology was determined by applying propagation and Otsu adaptive thresholding on the Phalloidin image channel. Cells touching the border of the image were filtered out of the dataset. Missegmentation artifacts were removed by applying an arbitrary threshold on nuclei and cell area. After background correction, the intensity values of the target of interest were calculated inside the nuclear area. The imaging software Fiji was used for image visualization.^[^ ^98^ ^]^

### Random Forest Classification to Associate Nuclei Morphology with Micro‐Topography Design Features

To identify surface design parameters that influence nuclei morphology, the top 200 micro‐topographies inducing either the highest and lowest statistically significant cell morphological parameters were selected and defined as positive or negative hits. This division in two classes allowed the use of a random forest binary classification for creating a predictive model associating micro‐topographical design parameters with nuclei size or other morphological parameters. For this, the measured value of each morphological parameter (e.g., nuclei area) was measured from each cell cultured on the micro‐topography. The random forest algorithm was run in R version 3.3.3.^[^ ^99^ ^]^ The accuracy of the model was represented by the receiver operating curve (ROC), which illustrates the performance of the binary classifier by plotting the true positive rate, against the false positive rate, at various threshold settings. In order to have a training set for testing the accuracy of the model, the data set was split into 2 parts. The first part contained 75% of the data and was used for model training and the remaining 25% was used for model testing. The models were trained with 10‐fold cross validation in “caret” package version 6.0.^[^ ^100^ ^]^ Receiver operating curve curves and the scatter plots separating high and low micro‐topographical hits were visualized through package “ggplot2”.^[^ ^101^ ^]^

### MSC Osteogenic and Adipogenic Differentiation

Differentiation of AD‐hMSCs toward the osteogenic lineage was achieved by seeding AD‐hMSCs at a density of 5 × 10^3^ cells/cm^2^. After 24 h, the medium was changed with either a control or mineralization medium. The mineralization media was basic media supplemented with 10% v/v FBS, 10 U/mL Penicillin/Streptomycin, with 10 nm dexamethasone (Sigma) and 10 mm *β*‐glycerol phosphate (Sigma), while control medium includes the same components except for dexamethasone. The media was refreshed every 2–3 days, and after 21 days, cells were fixed overnight at 4 °C with 4% formaldehyde (VWR) in PBS. Afterward, osteogenesis was assessed through staining mineralized deposits with a 2% Alizarin Red solution (pH = 4.2) for 2 min. Excess staining was washed off with demineralized water.

Differentiation of AD‐hMSCs toward the adipogenic lineage was achieved by seeding AD‐hMSCs at a density of 15 × 10^3^ cells/cm^2^. After 24 h, the medium was replaced with either a control or adipogenic medium. The adipogenic media consist of basic media supplemented with 10% v/v FBS, 10 U/mL Penicillin/Streptomycin, 0.5 mm 3‐isobutyl‐1‐methylxanthine (Sigma), 0.2 mm indomethacin (Sigma), 10 µg mL^−1^ Insulin (Sigma), and 1 µm dexamethasone (Sigma). The control medium consisted solely of basic media with 10% v/v FBS and 10 U/mL Penicillin/Streptomycin. The media was refreshed every 2–3 days, and after 21 days, cells were fixed overnight at 4 °C with 3.7% formaldehyde (VWR), 0.01 g mL^−1^ CaCl_2_.2H_2_O (Merck) in PBS. Afterward, adipogenesis was assessed by rinsing the fixation solution with demineralized water, and subsequently incubating the substrates in a 60% v/v 2‐propanol (VWR) for 5 min. Fat droplets were stained through a freshly filtered solution of 0.3% w/v Oil Red O dissolved in 60% v/v 2‐propanol (VWR). Afterward, the substrates were washed in triplicate with demineralized water.

### Statistical Analysis

Statistical analysis was performed with GraphPad Prism 9.4.1 (GraphPad Prism Software Incl. San Diego, USA). For image analysis, a *t*‐test was performed to evaluate significant differences between intensity levels of cells cultured on a flat surface compared to cells cultured on PS‐1018. In case multiple surface micro‐topographies were used, a one‐way ANOVA was applied. For histone mass‐spectrometry, a one‐way ANOVA test was applied to determine significant differences in histone levels between micro‐topography PS‐1018 and the flat surface.

## Conflict of Interest

The authors declare no conflict of interest.

## Supporting information

Supporting Information

## Data Availability

The data that support the findings of this study are openly available in DataverseNL at https://doi.org/10.34894/S2QX6C, reference number 0.
