# Supplementary material for: Micro‐Topographies Induce Epigenetic Reprogramming and Quiescence in Human Mesenchymal Stem Cells
Source: Adv Sci (Weinh). 2022 Nov 22;10(1):2203880. doi: 10.1002/advs.202203880 (PMC9811462; doi:10.1002/advs.202203880)
Supplement: Supplementary file 1 — Supporting Information [file ADVS-10-2203880-s001.pdf]

ADVANCED SCIENCE
Open Access

Supporting Information

for *Adv. Sci.*, DOI 10.1002/advs.202203880

Micro-Topographies Induce Epigenetic Reprogramming and Quiescence in Human Mesenchymal Stem Cells

*Steven Vermeulen, Bart Van Puyvelde, Laura Bengtsson del Barrio, Ruben Almey, Bernard K. van der Veer, Dieter Deforce, Maarten Dhaenens and Jan de Boer**

Supporting Information

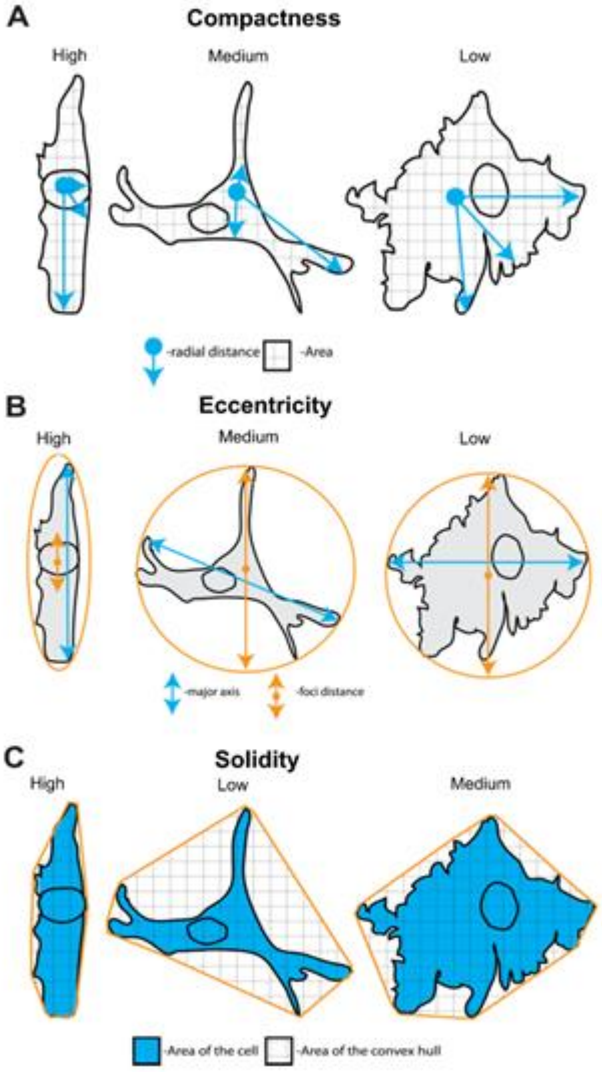

Figure S1: Schematic illustrations of the morphological parameters compactness, eccentricity, and solidity. A-B) Elongated cells exhibit high compactness and eccentricity values. **C)** Branched cells typically exhibit low solidity values.

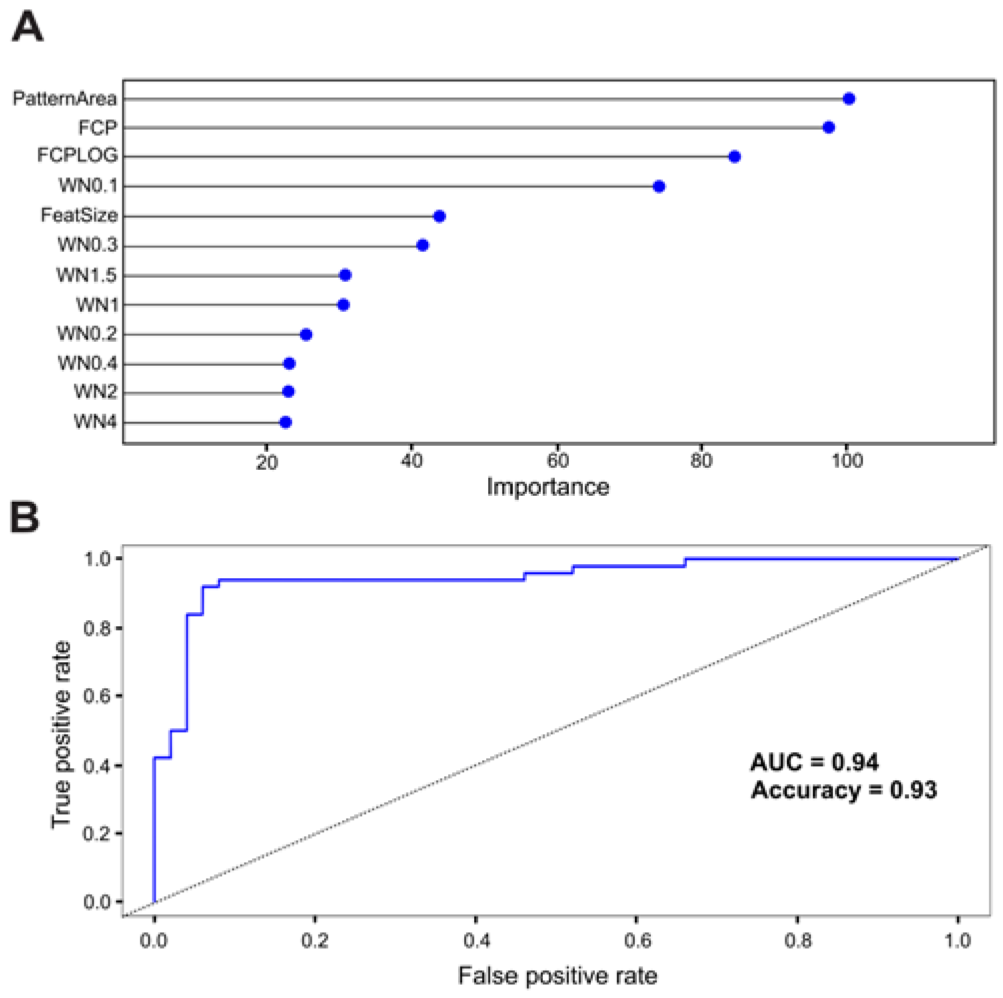

Figure S2: Random forest algorithms parameters. A) Random forest algorithms associate pattern area, FCP, FCPLOG, and WN0.1 as the most important feature parameters for determining the nuclear area. **B)** These parameters could be predicted with a high area under the curve (AUC) of 0.94, and an accuracy of 0.93.

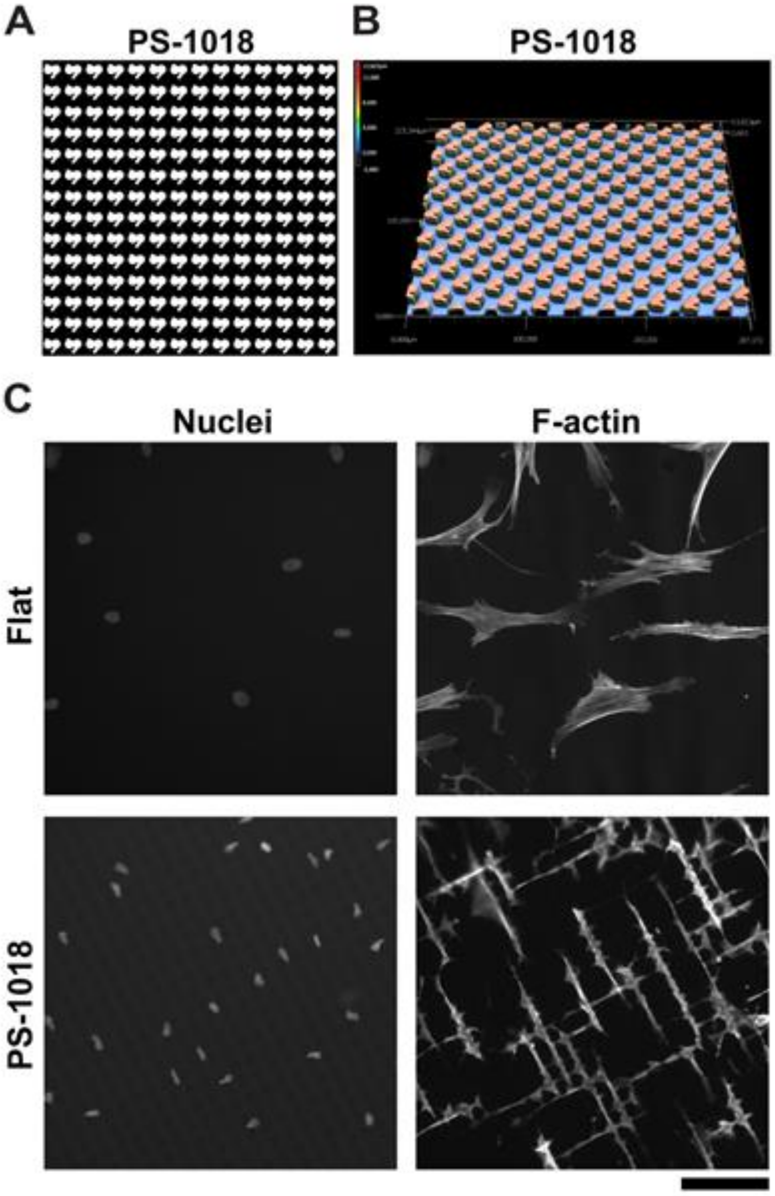

Figure S3: Surface PS-1018 induces elongated morphological characteristics and a reduction in cell and nuclear size. A) *In silico* design of the PS-1018 surface. B) Profilometric surface representation of surface PS-1018. **C)** MSCs cultured on the PS-1018 surface exhibit elongated characteristics and a mild reduction in cell and nuclear size. F-actin stained with phalloidin and nuclei counterstained with Hoechst33258. Scale bar represents 100 µm.

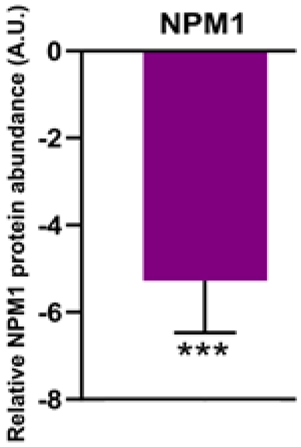

Figure S4: Proteomics data from the histone fraction reveals a sharp decrease of NPM1 in MSCs culture on the PS-1018 surface (n=6). Bar plots represents mean with error bars representing SEM.

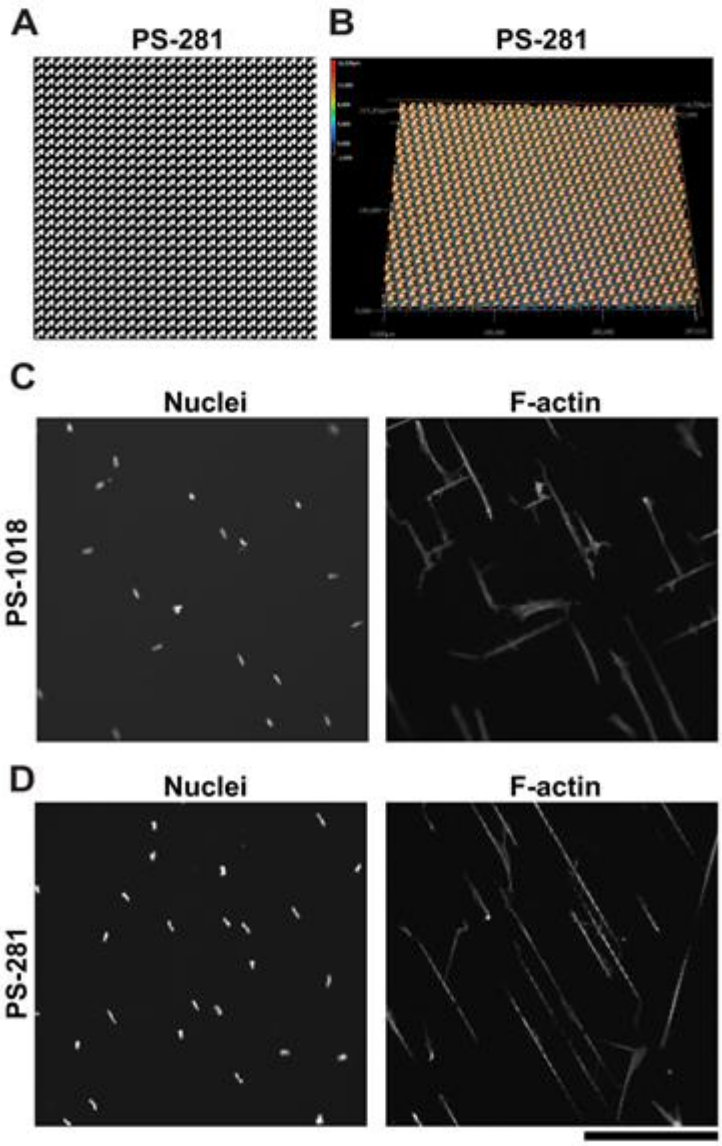

Figure S5: Comparison of morphological characteristics of MSCs cultured on the PS-1018 and PS-281 surface. A) Morphological characteristics of MSCs culture on the PS-1018 platform. **B)** Profilometric surface representation of surface PS-281. **C)** Morphological characteristics of MSCs culture on the PS-281 platform. F-actin stained with phalloidin and nuclei counterstained with Hoechst33258. Scale bar represents 200 µm.

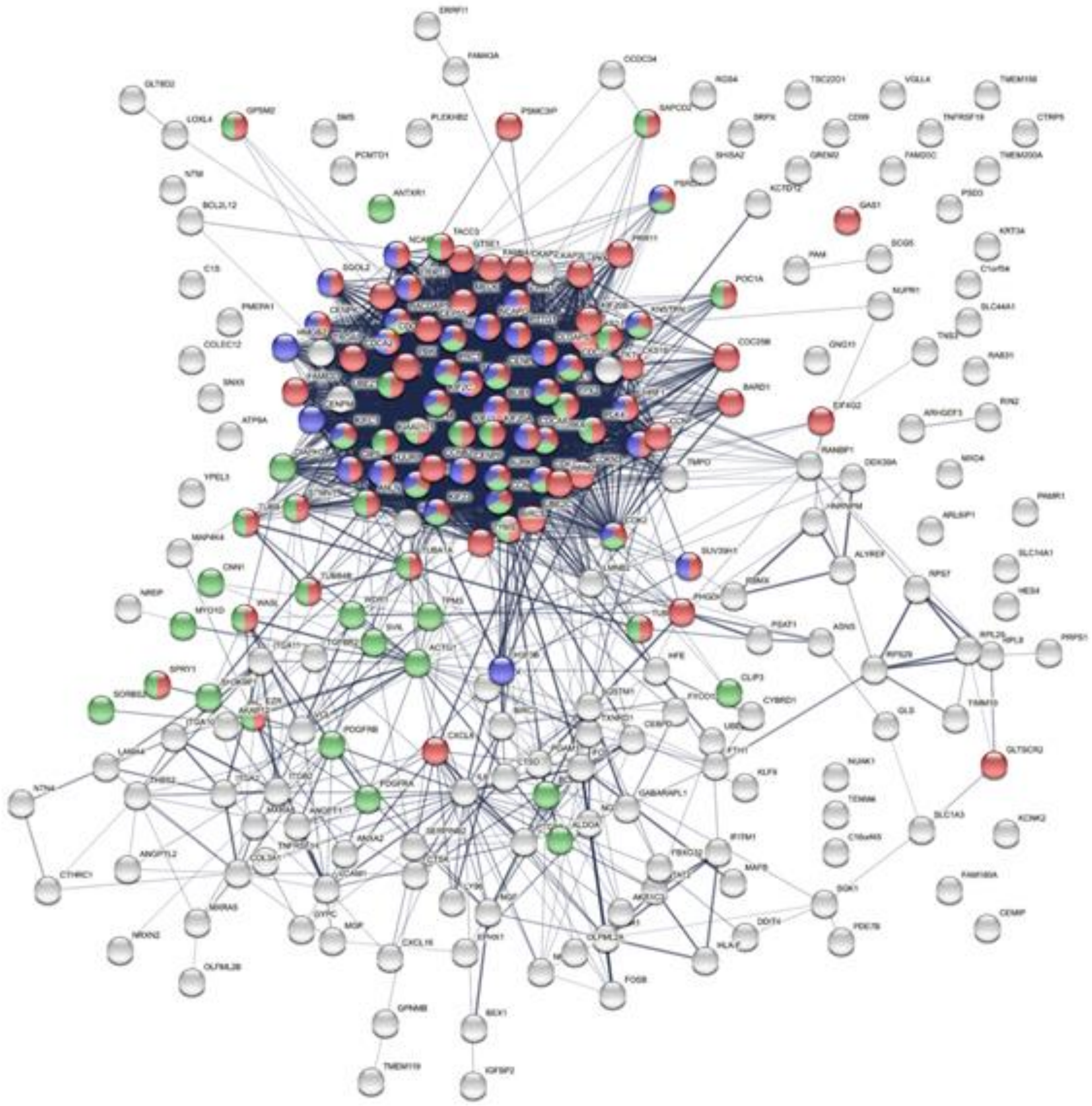

Figure S6: STRING network analysis of MSCs grown on surface PS-281. Highlighted GO terms are the cell cycle (GO:0007049; red), cytoskeletal organization (GO:0007010; green), and chromosome organization (GO:0051276; blue).

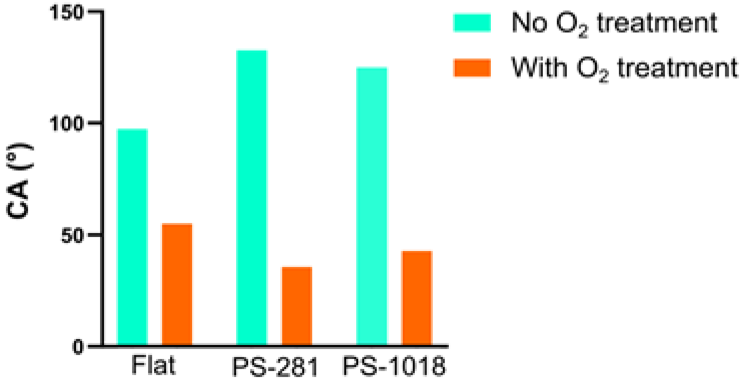

Figure S7: CA measurements of surfaces PS-281 and PS-1018 before and after plasma oxygen treatment.

Table Legends Supporting Information

Table S1: Description of the micro-topographical feature parameters.

Feature parameter	***Description***
Feature size	Side length of the feature area (10 µm, 20 µm or 28 µm).
FCP	Fraction of the feature area covered by primitives.
FCP LOG	Natural log of FCP/1-FCP.
Pattern area	The product of the feature size and the FCP.
Num Circ	Number of circles in the feature.
Num Tri	Number of triangles in the feature.
Num Line	Number of lines in the feature.
Circ Diam	The diameter of the circle(s) in the feature area.
Line Len	The length of the line(s) in the feature area.
TriSize	Shortest side length of a triangle primitive.
DC	The density of the circle primitives in a feature. Calculated by dividing the total number of circles by the feature size of the feature.
DT	The density of the triangle primitives in a feature. Calculated by dividing the total number of circles by the feature size of the feature.
DL	The density of the line primitives in a feature. Calculated by dividing the total number of circles by the feature size of the feature.
CA	The product of the density of circle primitives (DT) in a feature and the area of the circle primitive.
TA	The product of the density of triangle primitives (DC) in a feature and the area of a triangle primitive.
LA	The product of the density of line primitives (DL) in a feature and the area of a line primitive.
CCD	CCD is defined as the number of times a primitive is encountered starting over the diagonal from the bottom left corner to the top right corner divided by the length of this diagonal in µm.
WN ***X***	A two-dimensional discrete Fourier transformation representing the elements of the discretization of the feature as a sum of sinusoids with different wavenumbers. Eleven variables WN**X** with values ranging from 0.1 to 4 were created. These variables represent the fraction of the total energy that is present in sinusoids with wavenumber **X**. .E.g. a high value for WN**0.1** represents features containing relatively much energy in sinusoids with a low wavenumber.

Table S2: Description of the morphological parameters extracted from CellProfiler.

Morphological parameter	*Description*
Area	The number of pixels in the region.
Perimeter	The total number of pixels around the boundary of each region in the image.
FormFactor	Calculated as 4*π*Area/Perimeter2. Equals 1 for a perfectly circular object.
Solidity	Solidity: The proportion of the pixels in the convex hull that are also in the object, i.e. ObjectArea/ConvexHullArea. Equals 1 for a solid object (i.e., one with no holes or has a concave boundary), or <1 for an object with holes or possessing a convex/irregular boundary.
Extent	Extent: The proportion of the pixels in the bounding box that are also in the region. Computed as the Area divided by the area of the bounding box.
EulerNumber	EulerNumber: The number of objects in the region minus the number of holes in those objects, assuming 8-connectivity.
Center_X, Center_Y	Center_X, Center_Y: The x- and y-coordinates of the point farthest away from any object edge. Note that this is not the same as the Location-X and -Y measurements produced by the Identify modules.
Eccentricity	Eccentricity: The eccentricity of the ellipse that has the same second-moments as the region. The eccentricity is the ratio of the distance between the foci of the ellipse and its major axis length. The value is between 0 and 1. (0 and 1 are degenerate cases; an ellipse whose eccentricity is 0 is actually a circle, while an ellipse whose eccentricity is 1 is a line segment.)
MajorAxis Length	MajorAxisLength: The length (in pixels) of the major axis of the ellipse that has the same normalized second central moments as the region.
MinorAxis Length	MinorAxisLength: The length (in pixels) of the minor axis of the ellipse that has the same normalized second central moments as the region.
Orientation	Orientation: The angle (in degrees ranging from -90 to 90 degrees) between the x-axis and the major axis of the ellipse that has the same second-moments as the region.
Compactness	Compactness: The variance of the radial distance of the object's pixels from the centroid divided by the area.
MaximumRadius	MaximumRadius: The maximum distance of any pixel in the object to the closest pixel outside of the object. For skinny objects, this is 1/2 of the maximum width of the object.
MedianRadius	MedianRadius: The median distance of any pixel in the object to the closest pixel outside of the object.
MeanRadius	MeanRadius: The mean distance of any pixel in the object to the closest pixel outside of the object.
MinFeretDiameter, MaxFeretDiameter	MinFeretDiameter, MaxFeretDiameter: The Feret diameter is the distance between two parallel lines tangent on either side of the object (imagine taking a caliper and measuring the object at various angles). The minimum and maximum Feret diameters are the smallest and largest possible diameters, rotating the calipers along all possible angles.

Table S3: List of statistically significant altered levels of ribosome proteins derived from the histone protein fraction found in MSCs cultured on the PS-1018 micro-topography compared to MSCs cultured on a flat polystyrene surface.

Protein	Fold change	Adjusted p value
**RPS20**	-9.713559075	4.20073E-08
**RPS15**	-2.584705661	9.64313E-08
**RPL31**	-3.340351678	1.50076E-07
**RPS10**	-7.012845771	1.65902E-07
**RPS17**	-2.143546925	4.04207E-07
**RPL24**	-2.173469725	5.40648E-07
**RPL8**	-1.914542916	7.18924E-07
**RPL4**	-4.169863043	3.67887E-06

**RPL23**	-2.445280555	9.91608E-06
**RPL32**	-1.699369998	4.60461E-05
**RPL7**	-3.160165247	0.000141628
**RPL11**	-2.265767771	0.001355108
**RPL13A**	-1.510472586	0.02376715
**RPS26**	1.615521555	8.25825E-07
**MRPL12**	1.675974269	0.000141628

Table S4: List of statistically significant genes associated with the GO term "Chromosome organization" found in MSCs cultured on the PS-281 micro-topography compared to MSCs cultured on a flat polystyrene surface.

Gene	Fold change	Adjusted P value
**AURKA**	-2.091718218	2.60E-07
**AURKB**	-1.719497282	9.54E-08
**BUB1**	-1.660025981	1.75E-06
**CCNA2**	-1.896637688	1.52E-06
**CCNB1**	-1.594835947	1.47E-05
**CDC20**	-2.435905142	2.64E-09
**CDCA5**	-1.668699785	4.47E-08
**CDCA8**	-1.876849383	3.79E-10
**CENPA**	-1.75120909	4.56E-09
**CENPE**	-1.685934433	0.000114677
**CENPF**	-1.579788566	4.87E-07
**CENPK**	-1.516162276	1.79E-06
**DLGAP5**	-1.881806854	2.88E-07
**FEN1**	-1.503686051	0.000222228
**HJURP**	-1.699995916	1.23E-06
**KIF2C**	-1.714288742	2.36E-08
**KIFC1**	-1.540762269	1.56E-05
**KNSTRN**	-1.770854986	2.95E-07
**NCAPD2**	-1.547107091	2.98E-05
**NCAPG**	-1.72423968	2.27E-06
**NUSAP1**	-1.619281343	5.20E-07
**PRC1**	-1.795554027	5.24E-08
**PTTG1**	-1.790873828	3.02E-06
**PTTG3P**	-1.69217114	1.02E-05
**SUV39H1**	-1.521189184	4.06E-07
**TACC3**	-1.773775699	7.87E-08
**TOP2A**	-1.890114339	2.42E-08
**TRIP13**	-1.696751736	1.03E-08
**TTK**	-1.790995567	1.00E-07

Table S5: List of statistically significant genes associated with the GO term "Ribosome" found in MSCs cultured on the PS-281 micro-topography compared to MSCs cultured on a flat polystyrene surface.

Gene	Fold change	Adjusted P value
**MRPL12**	-1.406683775	0.001400768
**MRPL17**	-1.21742841	0.000942501
**MRPL24**	-1.241007649	0.031874499
**MRPL37**	-1.239565947	0.007857687
**MRPL41**	-1.318500185	0.000693718
**MRPS17**	-1.222295565	0.007067806
**MRPS34**	-1.209007981	0.010980501
**RPL13A**	1.454161194	5.23E-06
**RPL29**	-1.561319261	6.27E-07
**RPL39L**	-1.37208455	0.004020293
**RPL8**	-1.405264644	0.002619706
**RPS7**	-1.513616837	3.47E-06
**RSL24D1**	1.261315	0.000104355

Table S6: List of statistically significant genes associated with the GO term "Nucleolus" found in MSCs cultured on the PS-281 micro-topography compared to MSCs cultured on a flat polystyrene surface.

Gene	Fold change	Adjusted P value
**AGPAT5**	-1.23424	0.006791623
**AGPS**	-1.29413	0.001469657
**ANKRD1**	-1.44564	0.000829536
**APEX2**	-1.2191	0.000601447
**ARHGAP32**	1.316368	0.000116824
**BOP1**	-1.24794	0.003681358
**BRIX1**	-1.30135	0.007081602
**C1QBP**	-1.30221	0.000418976
**CBX5**	-1.42594	0.002199217
**CCDC86**	-1.33755	0.008128492
**CDCA8**	-1.87685	3.79E-10
**CENPW**	-1.41647	0.000495718
**CEP85**	-1.29501	0.000119162
**CHTOP**	-1.21201	0.002392754
**CKAP5**	-1.30958	0.002797629
**DDX21**	-1.215	0.021775982
**DDX23**	-1.27978	0.008003187
**DEK**	-1.22256	0.037152943
**DKC1**	-1.23638	0.019334002
**EBNA1BP2**	-1.63608	6.13E-05
**EMG1**	-1.44314	0.000157167
**EXOSC10**	-1.20401	0.021609182
**EXOSC2**	-1.22719	0.000202193
**EXOSC3**	-1.34736	0.000100659
**EXOSC9**	-1.2924	0.01991115
**EZR**	-1.90749	5.10E-08
**FANCG**	-1.45606	4.47E-08
**FBL**	-1.2171	0.013273688
**FEN1**	-1.50369	0.000222228
**GEMIN4**	-1.2013	0.007418662
**GRWD1**	-1.22237	0.001031654
**GTPBP4**	-1.26569	0.000777332
**HEATR1**	-1.20649	0.005746029
**HJURP**	-1.7	1.23E-06
**IFI16**	1.294665	0.016392153

**ILF2**	-1.23449	0.0093596
**IMP4**	-1.25667	0.0004327
**IPO5**	-1.3763	6.33E-05
**ITPR3**	-1.24381	0.004242198
**KIF20B**	-1.78369	7.13E-09
**LDB2**	1.435822	2.82E-06
**LDOC1**	1.235773	0.038071069
**LRWD1**	-1.2924	0.000496256
**LYAR**	-1.41404	0.000279451
**MAD2L2**	-1.23307	0.003585635
**MKI67**	-1.32014	2.53E-06
**MRTO4**	-1.41963	4.54E-06
**NCL**	-1.31909	0.008876398
**NEK2**	-1.27554	0.002580169
**NFIB**	1.237316	0.026220771
**NIP7**	-1.22819	0.002157357
**NOL11**	-1.24611	0.000775498
**NOL7**	-1.23548	0.027566807
**NOLC1**	-1.20916	0.000392555
**NOP16**	-1.28412	2.56E-05
**NOP58**	-1.2044	0.00856652
**NUAK1**	1.424032	4.30E-05
**NUSAP1**	-1.61928	5.20E-07
**OXR1**	1.325549	8.68E-05
**PA2G4**	-1.29526	0.018397326
**PARP1**	-1.20698	6.36E-05
**PKMYT1**	-1.39411	9.84E-05
**PLK4**	-1.558	1.54E-05
**POLR1E**	-1.25779	0.004892785
**PPP1CC**	-1.36098	0.005527594
**PTBP1**	-1.24056	0.000753763
**PTPN13**	1.247282	0.002398262
**RAN**	-1.2582	0.008022809
**RBL2**	1.280319	0.002663038
**RBM14**	-1.23416	0.008547382
**RGS2**	1.240103	0.001162108
**RNMT**	1.249489	0.000948666
**RPF2**	-1.24521	0.005621567
**RPL13A**	1.454161	5.23E-06
**RPS7**	-1.51362	3.47E-06
**RRP36**	-1.21761	0.001526585
**RRP7A**	-1.3359	3.15E-05
**RRS1**	-1.26965	0.001383549
**RSL24D1**	1.261315	0.000104355
**SAMD4A**	-1.27393	6.75E-05
**SAPCD2**	-1.44714	7.90E-07
**SNAPC1**	-1.21023	0.015217737
**SPC24**	-1.46663	4.71E-05
**SPG11**	1.207854	0.011284876
**SPTBN1**	-1.22595	0.024730934
**SUV39H1**	-1.52119	4.06E-07
**TOP2A**	-1.89011	2.42E-08
**TYMS**	-1.60623	3.31E-06
**UBE2T**	-1.56621	4.59E-06
**UCHL5**	-1.26101	0.00010053
**VRK1**	-1.26584	0.000154578
**XPC**	1.261586	0.005568026
**YPEL2**	1.205318	0.000159054
**YPEL3**	1.516852	2.89E-07

Table S7: List of statistically significant genes associated with the GO term “histone methyltransferase activity” found in MSCs cultured on the PS-281 micro-topography compared to MSCs cultured on a flat polystyrene surface.

Gene	Fold change	Adjusted P value
**EZH2**	-1.23905927	0.006015
**FBL**	-1.217101087	0.013274
**SUV39H1**	-1.521189184	4.06E-07
